# Supplementary material for: Microbiome and Microbial Pure Culture Study Reveal Commensal Microorganisms Alleviate Salmonella enterica Serovar Pullorum Infection in Chickens
Source: Microorganisms. 2024 Aug 23;12(9):1743. doi: 10.3390/microorganisms12091743 (PMC11434425; doi:10.3390/microorganisms12091743)
Supplement: Supplementary file 1 [file microorganisms-12-01743-s001.zip › microorganisms-3164209-supplementary.pdf]

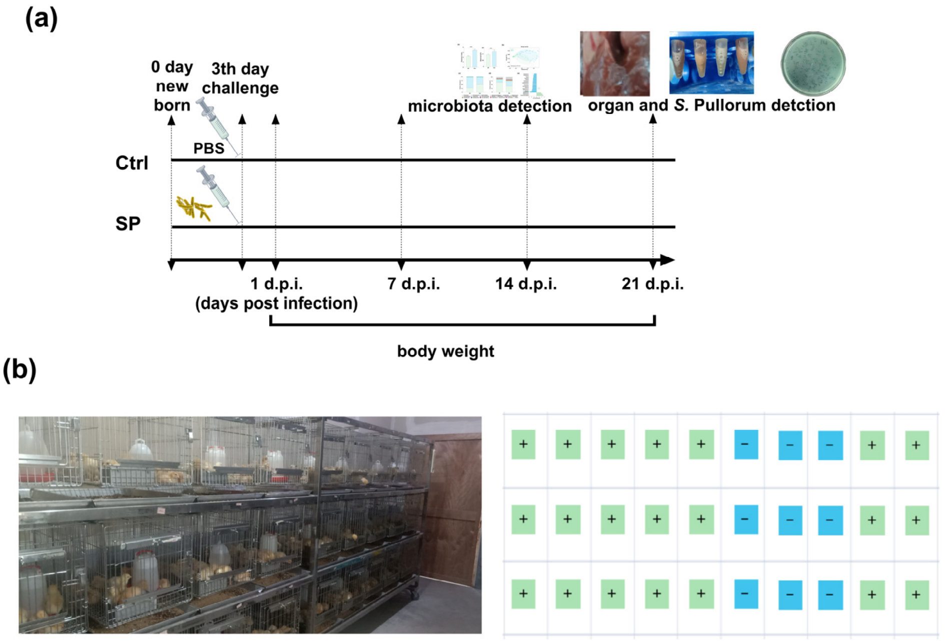

Supplementary Figure S1. Challenge with *S.* Pullorum in chicken (a) Flow chart of *S.* Pullorum Challenge. (b) Field photos and simulation diagram of chick rearing cages, "+" indicates the cages of infected chickens (green), and "-" indicates the cages of non-infected chickens (blue) and 353 infected chickens were raised in 21 cages (green) respectively, while 151 non-infected chickens were raised in 9 cages (blue).

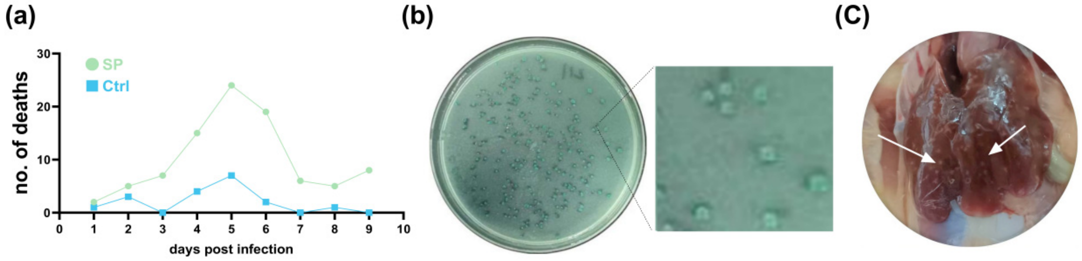

Supplementary Figure S2 The effects of *S.* Pullorum challenge on chicken performance. (a) Number of deaths following *S.* Pullorum challenge over days. (b) *S.* Pullorum load measured by plate counting with BSA selective medium, each green dot in the culture dish represents a colony of *S.* Pullorum. (c) Pathological alteration of liver in chicken infected with *S.* Pullorum.

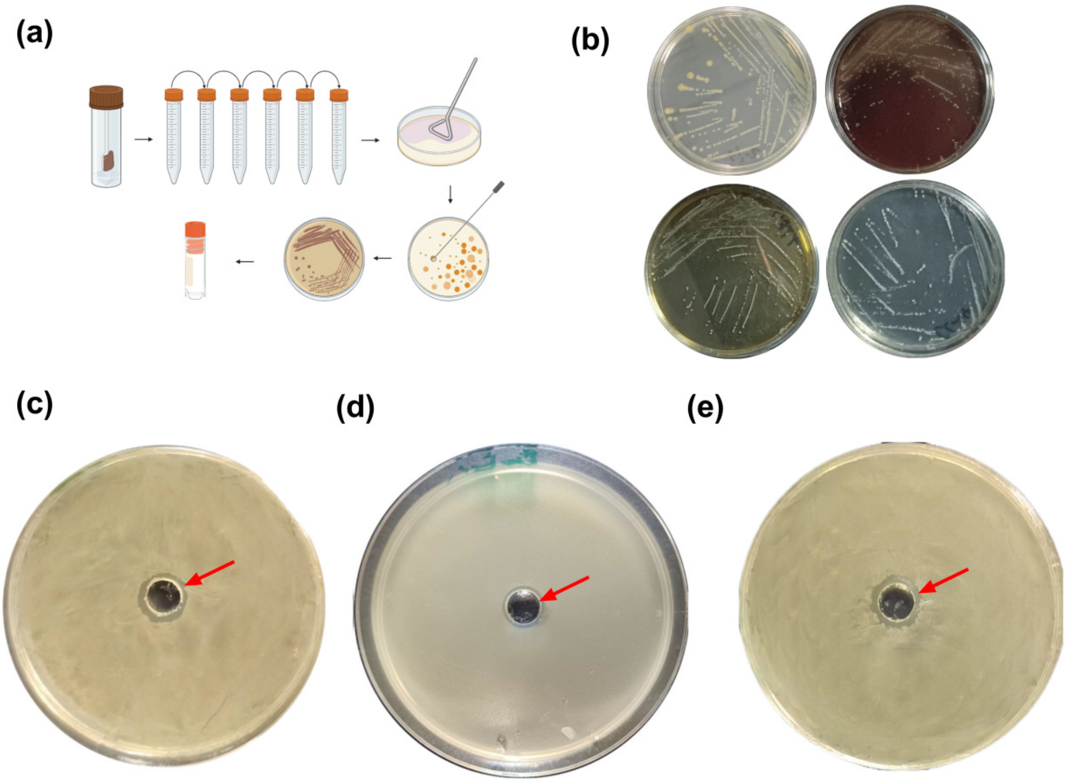

Supplementary Figure S3. Microorganisms isolated from chicken feces for antibacterial test (a) Flow chart of *S.* Pullorum related microorganisms isolation. (b) Different types of culture media are used to isolate different target microorganisms. The antibacterial ability of *Bifidobacterium pseudolongum* (c), *Streptococcus equi* (d) and *Lacticaseibacillus paracasei* (e) against *S.* Pullorum was measured by the agar dilution method in vitro, the red arrow points to the inhibition zone.

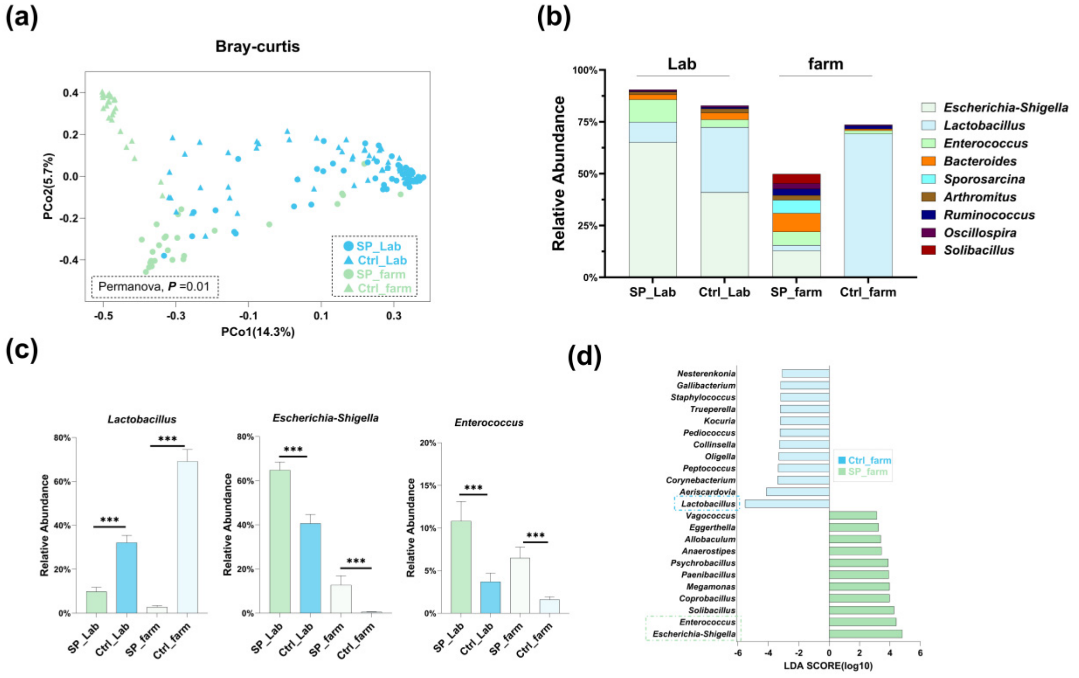

Supplementary Figure S4. Gut microbiota profile of *S.* Pullorum infection chicken in farm (a) Principal coordinate analysis of chicken gut microbiota in Lab and farm with Bray-Curtis distance. (b) Relative abundance of gut bacterial

taxonomic compositions at general taxonomic levels. (c) genus with significant differences(d) Lefse analysis reveal biomarkers of chicken gut microbiota in farm.

Supplementary Table S1. The types and quantities of isolated *S.* Pullorum infection microorganisms

Species	No.
Lactiplantibacillus plantarum	9
Limosilactobacillus reuteri	8
Lactobacillus pentosus	5
Lacticaseibacillus rhamnosus	3
Lacticaseibacillus paracasei	2
Lactobacillus paraplantarum	1
Lactobacillus johnsonii	1
Pediococcus pentosaceus	4
Lactococcus lactis	2
Clostridium butyricum	1
Bifidobacterium pseudolongum	1
Streptococcus equi	1
